# Boric acid transport activity of human aquaporins expressed in *Xenopus* oocytes

**DOI:** 10.14814/phy2.15164

**Published:** 2022-01-10

**Authors:** Kazutaka Ushio, Erika Watanabe, Takehiro Kamiya, Ayumi Nagashima, Tadaomi Furuta, Genki Imaizumi, Toru Fujiwara, Michael F. Romero, Akira Kato

**Affiliations:** ^1^ School of Life Science and Technology Tokyo Institute of Technology Yokohama Japan; ^2^ Department of Applied Biological Chemistry Graduate School of Agricultural and Life Sciences The University of Tokyo Tokyo Japan; ^3^ Department of Physiology and Biomedical Engineering Mayo Clinic College of Medicine & Science Rochester Minnesota USA; ^4^ Nephrology and Hypertension Mayo Clinic College of Medicine & Science Rochester Minnesota USA; ^5^ O’Brien Urology Research Center Mayo Clinic College of Medicine & Science Rochester Minnesota USA; ^6^ Center for Biological Resources and Informatics Tokyo Institute of Technology Yokohama Japan

**Keywords:** aquaglyceroporin, aquaporin, boric acid channel, electrophysiology, *Xenopus* oocyte

## Abstract

Boric acid is a vital micronutrient that is toxic at high concentrations in animals. However, the mechanisms underlying boric acid transport in animal cells remain unclear. To identify the plasma membrane boric acid channels in animals, we analyzed the function of human aquaporins (AQPs), which are homologous to the nodulin‐like intrinsic protein family of plant boric acid channels. When human AQPs were expressed in *Xenopus laevis* oocytes, the results of the swelling assay showed that boric acid permeability significantly increased in oocytes expressing AQP3, 7, 8, 9, and 10, but not in those expressing AQP1, 2, 4, and 5. The boric acid influxes of these oocytes were also confirmed by elemental quantification. Electrophysiological analysis using a pH microelectrode showed that these AQPs transported boric acid (B(OH)_3_) but not borate ions (B(OH)_4_^–^). These results indicate that AQP3, 7, 8, 9, and 10 act as boric acid transport systems, likely as channels in humans.

## Graphical abstract

Boric acid transport activities of human aquaporins (AQPs) were analyzed by *Xenopus* oocyte swelling assay, elemental quantification, and electrophysiological analysis using a pH microelectrode. The results indicate that human AQP3, 7, 8, 9 and 10 act as boric acid channels. This is the first demonstration of identification of boric acid channels in mammals.

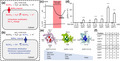

## 1 INTRODUCTION

Boron is an essential element in various organisms. The biological importance of boron was initially described in plants (Tanaka & Fujiwara, 2008; Warington, 1923). Boron deficiency influences the growth and expansion of plant organs, such as leaves, roots, flowers, fruits, and seeds, and boron availability is widely recognized as an important determinant of agricultural production (Blevins & Lukaszewski, 1998; Tanaka & Fujiwara, 2008). In mammals, the significance of boron has not been established; however, boron has many beneficial effects on human health and well‐being and is considered to be nutritionally important (Nielsen, 2014; Uluisik et al., 2018). Boron plays beneficial roles in bone growth and maintenance, hormone functions, brain function, amelioration of arthritis, and cancer risk reduction (Nielsen, 2014; Uluisik et al., 2018); however, the biochemical mechanisms of boron function remain unclear.

The mechanisms underlying boron transport were also initially described in plants. The lipid bilayer of biological membranes is permeable to boric acid; however, membrane transport proteins also significantly contribute to the transport of boric acid across the biological membrane (Dordas et al., 2000). Two boron transport systems have been isolated in plants: active transport by the BOR transporter and facilitated transport by nodulin‐like intrinsic protein (NIP) channels (Miwa & Fujiwara, 2010; Tanaka & Fujiwara, 2008). BOR1 was initially identified in *Arabidopsis* as a membrane protein required for growth under low boron conditions (Takano et al., 2002). Plant BOR1 is homologous to the mammalian solute carrier 4 (SLC4) family of bicarbonate transporters. The human homolog of BOR1 is SLC4A11 (Parker et al., 2001). Human SLC4A11 was initially characterized as an Na^+^‐coupled borate cotransporter NaBC1 (Park et al., 2004); however, recent studies by several groups have shown that mammalian SLC4A11 displays H^+^/OH^−^ transport in both an Na^+^‐independent and Na^+^‐coupled mode but does not transport borate (Kao et al., 2020; Loganathan et al., 2016; Myers et al., 2016; Ogando et al., 2013). Currently, in mammals and humans, no SLC4 family proteins are believed to transport borate. The NIP family is another type of boron transporter that belongs to the major intrinsic protein (MIP) family or aquaporin (AQP) water channel superfamily. NIP5;1 was initially identified as a boric acid channel required for boric acid uptake and normal growth in *Arabidopsis* (Takano et al., 2006). NIPs are divided into three subclasses (I–III), and NIP II proteins, including NIP5;1, have been characterized as boric acid channels.

The human AQP family consists of 13 members, AQP0–12, which belong to three subfamilies: orthodox or classical AQPs that selectively transport water; aquaglyceroporins that transport water and other small neutral solutes, such as glycerol and urea; and unorthodox or super AQPs, whose function is still uncertain (Agre et al., 2002; Azad et al., 2021; Borgnia et al., 1999). In humans, AQP3, 7, 9, and 10 are recognized as aquaglyceroporins. AQP3 is widely expressed in many organs, whereas AQP7, 9, and 10 are highly expressed in the adipose tissue, liver, and intestine, respectively. These aquaglyceroporins are involved in maintaining water homeostasis and play significant roles in glycerol and urea metabolism. The boric acid transport activities of AQPs have not yet been completely evaluated in animals (Hibuse et al., 2006; Laforenza et al., 2016; Litman et al., 2009). To determine whether AQPs act as boric acid channels in humans, we expressed human AQPs in *Xenopus laevis* oocytes and analyzed their activity through swelling assays, elemental quantification, and electrophysiology. The results indicated AQP3, 7, 8, 9, and 10 act as boric acid transport systems, perhaps channels (in keeping with water, glycerol, and urea membrane transit).

## 2 MATERIALS AND METHODS

### 2.1 Expression of human AQPs in *Xenopus* oocytes

Total RNA from human tissues, the brain, kidney, pancreas, liver, and small intestine was purchased from Clontech (Clontech‐Takara Bio). Five micrograms of total RNA was reverse transcribed using oligo(dT) primers and the SuperScript III First‐Strand Synthesis System (Thermo Fisher Scientific). Full‐length cDNAs of AQPs were isolated from the kidney (AQP1, 2, 3, 7), brain, (AQP4), lung (AQP5), pancreas (AQP8), liver (AQP9), and small intestine (AQP10) by RT‐PCR using primers designed based on the genomic database (Table 1). cDNAs were subcloned into pGEMHE (Liman et al., 1992) and sequenced to confirm the absence of PCR errors.

**TABLE 1:** List of primers used for polymerase chain reaction amplification

Gene	Accession	Direction	Sequence (5’ to 3’)	Derived tissue
AQP1	NM_198098	Forward	TGCCAGCATGGCCAGTGAAATCAAGAAGAA	Kidney
		Reverse	CTCTATTTGGGCTTCATCTCCACCCTGGAG	
AQP2	NM_000486	Forward	CCATGTGGGAGCTCCGCTCCATAGCCTTCT	Kidney
		Reverse	CTCAGGCCTTGGTACCCCGTGGCAGGCTCT	
AQP3	NM_004925	Forward	CCATGGGTCGACAGAAGGAGCTGGTGTCCC	Kidney
		Reverse	CTCAGATCTGCTCCTTGTGCTTCACATGGG	
AQP4	NM_001650	Forward	ATGAGTGACAGACCCACAGCAAGGCGGTGG	Brain
		Reverse	GTCATACTGAAGACAATACCTCTCCAGATT	
AQP5	NM_001651	Forward	ATGAAGAAGGAGGTGTGCTCCGTGGCCTTC	Lung
		Reverse	TCAGCGGGTGGTCAGCTCCATGGTCTTCTT	
AQP7	NM_001170	Forward	CCATGGTTCAAGCATCCGGGCACAGGCGGT	Kidney
		Reverse	CCTTAGAAGTGCTCTAGGGCCATGGATTCA	
AQP8	NM_001169	Forward	TGAGCAGATGTCTGGGGAGCAGACACCAAT	Pancreas
		Reverse	TCTTCACCTCGACTTTAGAATTAGGCGGGT	
AQP9	NM_020980	Forward	CGATGCAGCCTGAGGGAGCAGAAAAGGGAA	Liver
		Reverse	ACTACATGATGACACTGAGTTCATATTTCT	
AQP10	NM_080429	Forward	CCATGGTCTTCACTCAGGCCCCGGCTGAAA	Small intestine
		Reverse	TTCATAGCTTACACTCCAGCATCTGAGCTG	

*Xenopus laevis* oocytes were dissociated with collagenase as described previously (Romero et al., 1998) and injected with either 50 nl of water (control) or a solution containing cRNA at 0.5 ng/nl (25 ng/oocyte), using a Nanoject II injector (Drummond Scientific). Oocytes were incubated at 16°C in OR3 medium and studied for 3–4 days after injection. One liter of OR3 medium contained 0.7% w/v powdered Leibovitz L‐15 medium with L‐Glutamine (Thermo Fisher Scientific), 50 ml of 10,000 U penicillin and 10,000 U streptomycin solution in 0.9% NaCl (Sigma‐Aldrich), and 5 mM HEPES, pH 7.50, and the osmolarity was adjusted to 200 mosmol/kg with NaCl powder (Romero et al., 1998). All *Xenopus* care and oocyte harvest protocols were in accordance with the National Institutes of Health “Guide for the Care and Use of Laboratory Animals.” Frogs were housed and cared for in accordance with the approval of the Institutional Animal Care and Use Committee of the Mayo Clinic College of Medicine, and in accordance with a manual approved by the Institutional Animal Experiment Committee of the Tokyo Institute of Technology.

To analyze the effects of cRNA amounts injected into oocytes on their activity, oocytes were injected with either 50 nl of water (control) or a solution containing AQP9 cRNA at 0.5 ng/nl (25 ng/oocyte), 0.2 ng/nl (10 ng/oocyte), 0.08 ng/nl (4 ng/oocyte), 0.032 ng/nl (1.6 ng/oocyte), or 0.013 ng/nl (0.64 ng/oocyte), incubated at 16 °C in OR3 medium, and studied for 4 days after injection.

### 2.2 Swelling assays

All experiments were performed at room temperature (23°C). Swelling of oocytes was monitored using a stereo microscope (SZX9, Olympus) equipped with a charge‐coupled device (CCD) camera (DS‐Fi2, Nikon). Photographs of oocytes were taken every 30 s using the NIS‐Elements D software (Nikon). Oocyte volumes were calculated assuming a spherical geometry. Oocytes incubated in ND96 saline solution (96 mM NaCl, 2 mM KCl, 1 mM MgCl_2_, 1.8 mM CaCl_2_, and 5 mM HEPES, pH 7.5, ~200 mosmol/kg) were transferred to two times diluted ND96 (~100 mosmol/kg) for water transport assays (Takano et al., 2006). For boric acid, glycerol, or urea transport assays, oocytes were transferred to an isotonic solution containing ND96 supplemented with 180 mM boric acid, glycerol, or urea instead of NaCl to adjust the osmolarity to ~200 mosmol/kg.

Quantitative data with oocytes from at least two frogs are presented as the mean ± SEM. Whiskers indicate the minimum and maximum values excluding outliers. Values greater than 1.5 times the interquartile range are out of the box plot and considered as outliers. Changes in volume (nl/min) were compared among oocytes expressing AQPs and control oocytes, and the statistical significance was evaluated using the Kruskal–Wallis test followed by the Mann–Whitney *U* test applying the Bonferroni correction for multiple comparisons (*α* = 0.05); the statistical analyses were performed using GraphPad Prism software (Version 5, GraphPad).

The relationships between water, glycerol, urea, and boric acid permeability of oocytes were analyzed using the Pearson correlation. The average values of the results of the swelling assays of oocytes injected with 50 nl water or 25 ng cRNA for AQP1, 2, 3, 4, 5, 7, 8, 9, or 10 were calculated. Four datasets were prepared for the experiments using each solution: (i) the hypo‐osmotic solution, (ii) iso‐osmotic solution containing 180 mM glycerol, (iii) iso‐osmotic solution containing 180 mM urea, and (iv) iso‐osmotic solution containing 180 mM boric acid. The correlation between two of the four datasets was calculated by the Pearson correlation using Excel software (Version 2019, Microsoft).

Phloretin (Tokyo Chemical Industry) was dissolved in dimethyl sulfoxide (DMSO) to prepare a 100 mM stock solution. Oocytes injected with 25 ng cRNA for AQP9 were used to analyze the effect of phloretin. AQP9 oocytes were preincubated in ND96 solution containing 100 μM phloretin or 0.1% DMSO for 30 min, and then transferred to two times diluted ND96 or ND96 supplemented with 180 mM boric acid with 100 μM phloretin or 0.1% DMSO. Swelling of the oocytes was monitored as described above.

### 2.3 Ion‐selective microelectrode analysis

To measure the intracellular pH (pH_i_) of oocytes, H^+^ ion‐selective microelectrodes were prepared with an H^+^ ionophore I‐mixture B ion‐selective resin (Fluka Chemical) as previously described (Sciortino & Romero, 1999). pH_i_ was measured as the difference between the pH electrode and a KCl voltage electrode impaled into the oocyte using a two‐channel electrometer (FD223a, World Precision Instruments), and the membrane potential (*V*_m_) was measured as the difference between the KCl microelectrode and an extracellular calomel connected to a single electrometer (Electra 705, World Precision Instruments). pH electrodes were calibrated using pH 6.0 and 8.0 buffers (Wako Pure Chemical Industries), followed by a point calibration in ND96 (pH 7.5).

Oocytes injected with 50 nl water or 25 ng cRNA for AQP3, 4, 7, 8, 9, or 10 were used for the analyses. Oocytes were held on a nylon mesh in a chamber and perfused with solution. *V*_m_ and pH_i_ were constantly monitored and recorded at 0.1 Hz using an analog‐to‐digital converter (PowerLab 8/35, AD Instruments) and LabChart software (AD Instruments). Solutions containing 10 or 3 mM boric acid were prepared by substituting NaCl with boric acid. The osmolarity and pH of all media were adjusted to ~200 mOsm and 7.5, respectively.

To analyze the effect of phloretin, oocytes injected with 25 ng cRNA for AQP9 were used for the analyses. Oocytes expressing AQP9 were preincubated in ND96 solution containing 100 μM phloretin or 0.1% DMSO for 30 min, held on a nylon mesh in a chamber, and perfused with the same solution. *V*_m_ and pH_i_ were monitored, as described above. The solution was then replaced with ND96 solution containing 10 mM boric acid, 100 μM phloretin, or 0.1% DMSO.

Oocytes injected with 25 ng cRNA for AQP9 were used to analyze the effects of glycerol and urea. Oocytes expressing AQP9 were held on a nylon mesh in a chamber and perfused with ND96 solution. *V*_m_ and pH_i_ were monitored, as described above. The solution was then replaced with (i) ND96 solution containing 3 mM boric acid, (ii) ND96 solution containing 3 mM boric acid and 10 mM glycerol, and (iii) ND96 solution containing 3 mM boric acid and 10 mM urea. The osmolarity and pH of the media were adjusted to ~200 mOsm and 7.5, respectively.

Quantitative data with oocytes from at least two frogs are presented as the mean ± SEM. Values for ΔpHi/dt were compared between oocytes expressing AQPs and the same‐batch control oocytes, and the statistical significance (*p* < 0.05) was evaluated by the Welch's *t*‐test using GraphPad Prism software.

### 2.4 Quantitative determination of boron content by inductively coupled plasma mass spectrometry (ICP‐MS)

Oocytes injected with 50 nl water or 25 ng cRNA for AQP1, 3, 7, 8, 9, or 10 were placed in ND96 containing 10 mM boric acid for 10 min at 23°C. Each oocyte was washed with ND96 for several seconds and dried. Dried *Xenopus* oocytes were digested with concentrated nitric acid in Teflon tubes, and the residues were dissolved in 0.08 M nitric acid containing 5 µg/L Be. Concentrations of boron‐10 and boron‐11 were measured by ICP‐MS (Agilent 7800 ICP‐MS, Agilent Technologies) using Be as an internal standard, and the sum of boron‐10 and boron‐11 concentrations is presented as the B concentration (Takano et al., 2002).

Quantitative data with oocytes from at least two frogs are presented as the mean ± SEM. Values for boron content (nmol/oocyte) were compared among oocytes expressing AQPs and control oocytes, and the statistical significance was evaluated using the Kruskal–Wallis test followed by the Mann–Whitney *U* test, applying the Bonferroni correction for multiple comparisons (*α* = 0.05); these tests were performed using GraphPad Prism software.

### 2.5 Computational structural analysis of AQPs

The structures of AQP10 (PDB: 6F7H) (Gotfryd et al., 2018) and AQP2 (PDB: 4NEF) (Frick et al., 2014) were obtained from the Protein Data Bank (PDB, https://www.rcsb.org/), and the AQP8 model (Model: AlphaFold‐Q94778) was obtained from the AlphaFold Protein Structure Database (https://alphafold.ebi.ac.uk/) (Jumper et al., 2021). The pore sizes were estimated using MOLE online (https://mole.upol.cz/) (Pravda et al., 2018). The ar/R residue table was constructed based on multiple alignments by Clustal Omega (https://www.ebi.ac.uk/Tools/msa/clustalo/) (Sievers et al., 2011) using amino acid sequences of human AQP1 (NCBI protein database: NP_932766), 2 (NP_000477), 3 (NP_004916), 4 (NP_001641), 5 (NP_001642), 7 (NP_001161), 8 (NP_001160), 9 (NP_066190), and 10 (NP_536354).

## 3 RESULTS

### 3.1 Water, glycerol, and urea permeability of *Xenopus* oocytes expressing human AQPs

**FIGURE 1:**
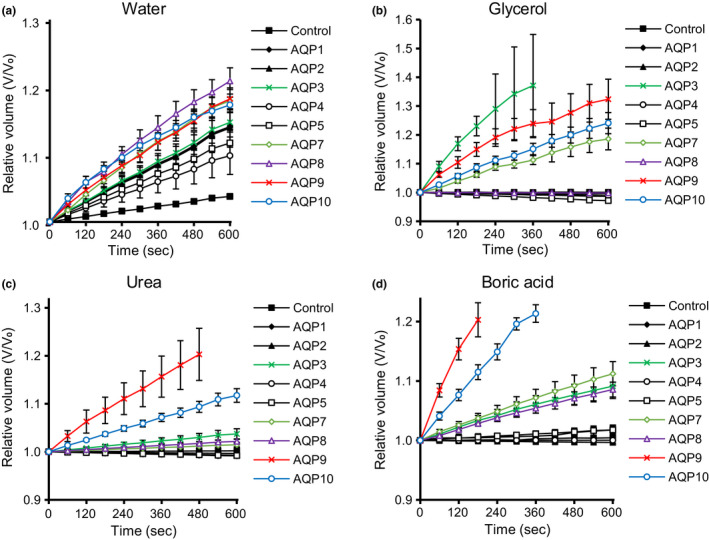
Increase in volume of AQP‐expressing and control oocytes by time (s). Oocytes in (a) hypo‐osmotic solution; (b) iso‐osmotic solution containing 180 mM glycerol; (c) urea; and (d) boric acid. Values are shown as mean ± SEM (*n* = 10–24)

**FIGURE 2:**
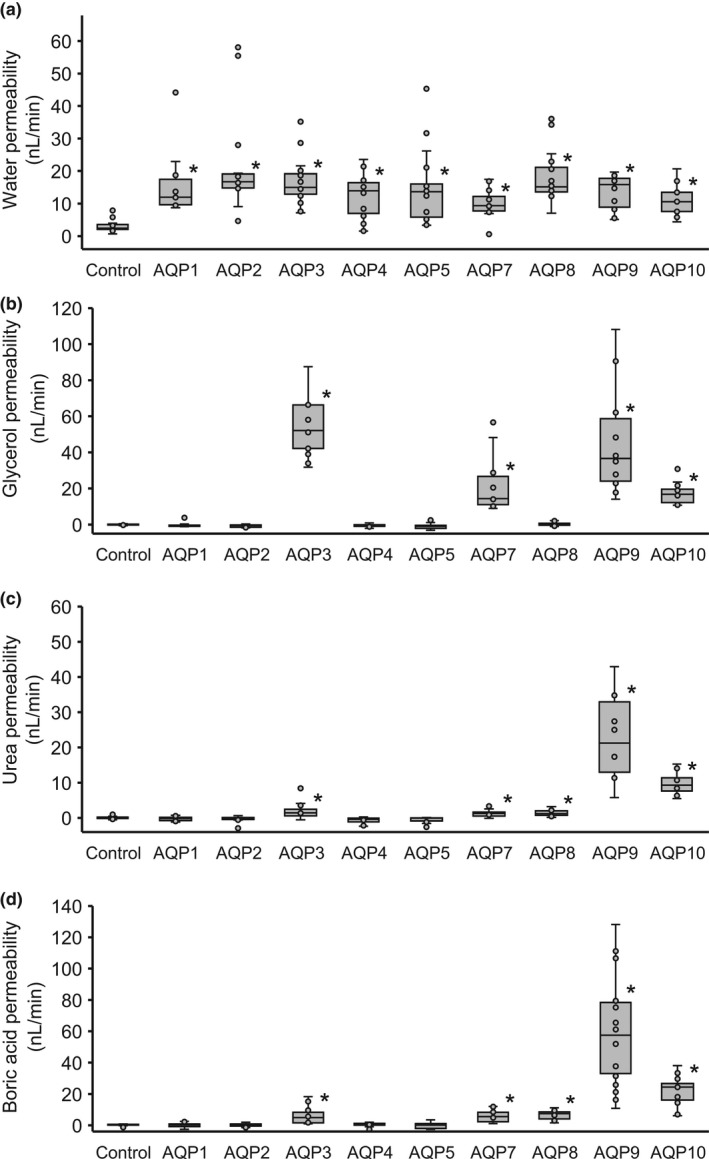
Water (a), glycerol (b), urea (c), and boric acid (d) transport activity of AQPs measured by oocyte swelling assays. The change in volume of oocytes expressing each AQP was compared with those of control oocytes. Values are presented as median (line), interquartile range (box), range (whiskers), and outliers (>1.5× interquartile range above upper quartile). Statistical significance was evaluated using the Kruskal–Wallis test followed by the Mann–Whitney *U* test; the Bonferroni correction was applied for multiple comparisons (*p < 0.05/9 = 0.0056)

We first analyzed the water permeability of oocytes expressing AQPs using a swelling assay to confirm the expression of AQPs in the plasma membrane of oocytes. The water permeability of oocytes was evaluated as the increase in volume when oocytes were exposed to a hypo‐osmotic solution. Oocytes expressing AQP1, 2, 3, 4, 5, 7, 8, 9, and 10 showed significant increase in cell volume in the hypo‐osmotic solution (*n* = 10 for AQP1, *p* < 0.0056; *n* = 14 for AQP2, *p* < 0.0056; *n* = 23 for AQP3, *p* < 0.0056; *n* = 19 for AQP4, *p* < 0.0056; *n* = 22 for AQP5, *p* < 0.0056; *n* = 18 for AQP7, *p* < 0.0056; *n* = 16 for AQP8, *p* < 0.0056; *n* = 14 for AQP9, *p* < 0.0056; *n* = 17 for AQP10, *p* < 0.0056; vs. control, *n* = 24 using the Mann–Whitney *U* test with Bonferroni correction *α* = 0.0056) (Figures 1a and 2a), demonstrating that these AQPs act as water channels in the plasma membrane of oocytes.

We next analyzed the glycerol and urea permeability of oocytes expressing AQPs through swelling assays with an iso‐osmotic solution containing glycerol or urea to confirm that our system showed results similar to those of previous studies. In the iso‐osmotic solution containing 180 mM glycerol, oocytes expressing AQP3, 7, 9, and 10 showed significant increase in cell volume (*n* = 13 for AQP3, *p* < 0.0056; *n* = 10 for AQP7, *p* < 0.0056; *n* = 10 for AQP9, *p* < 0.0056; *n* = 12 for AQP10, *p* < 0.0056; vs. control, *n* = 16 using the Mann–Whitney *U* test with Bonferroni correction α = 0.0056) (Figures 1b and 2b), indicating that these AQPs increased glycerol permeability. In the iso‐osmotic solution containing 180 mM urea, oocytes expressing AQP3, 7, 8, 9, and 10 showed significant increase in cell volume (*n* = 18 for AQP3, *p* < 0.0056; *n* = 15 for AQP7, *p* < 0.0056; *n* = 12 for AQP8, *p* < 0.0056; *n* = 10 for AQP9, *p* < 0.0056; *n* = 11 for AQP10, *p* < 0.0056; vs. control, *n* = 19 using the Mann–Whitney *U* test with Bonferroni correction *α* = 0.0056) (Figures 1c and 2c), indicating that these AQPs increased urea permeability. These results also confirmed that AQP1, 2, 4, and 5 increased water but not glycerol or urea permeability and can be categorized as “orthodox” or “classical” AQPs.

### 3.2 Boric acid permeability of *Xenopus* oocytes expressing human AQPs evaluated by swelling assays

The boric acid permeability of oocytes expressing AQPs was analyzed through swelling assays with an iso‐osmotic solution containing boric acid. In an iso‐osmotic solution containing 180 mM boric acid, oocytes expressing AQP3, 7, 8, 9, and 10 showed significant increase in cell volume (*n* = 20 for AQP3, *p* < 0.0056; *n* = 14 for AQP7, *p* < 0.0056; *n* = 19 for AQP8, *p* < 0.0056; *n* = 19 for AQP9, *p* < 0.0056; *n* = 11 for AQP10, *p* < 0.0056; vs. control, *n* = 51 using the Mann–Whitney *U* test with Bonferroni correction *α* = 0.0056) (Figures 1d and 2d). No swelling was observed during the immersion of the control, AQP1, 2, 4, and 5.

**FIGURE 3:**
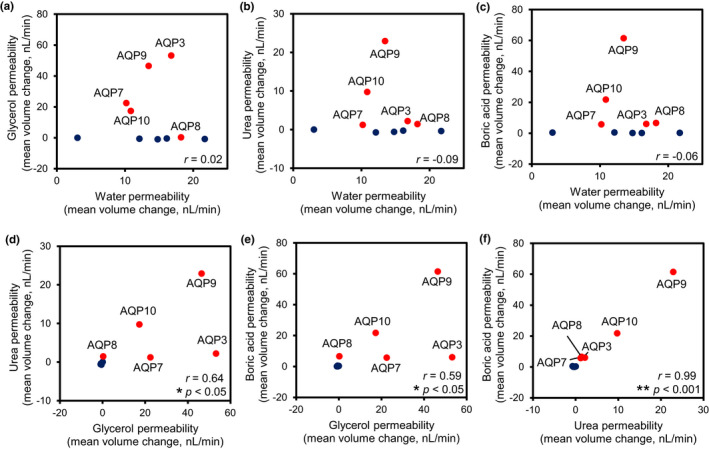
Scatter plots and Pearson correlation coefficient (*r*) between water, glycerol, urea, and boric acid permeabilities of oocytes expressing each AQP. (a–f) The average rates of volume changes in oocytes expressing boric acid‐permeable AQPs (AQP3, 7, 8, 9, and 10) were plotted in red, and those of oocytes expressing boric acid impermeable AQPs (AQP1, 2, 4, and 5) or water‐injected oocytes were plotted in navy blue. *p < 0.05; **p < 0.001; *n* = 10

To analyze the relationships between water, glycerol, urea, and boric acid permeability of oocytes expressing AQPs, the average values of the results of the swelling assays of each AQP were plotted and the strengths of the permeabilities were compared between substrates using the Pearson correlation (Figure 3). In Figure 3, the results for water‐injected or AQP1, 2, 3, 4, 5, 7, 8, 9, or 10‐expressing oocytes are plotted, and a total of 10 plots were analyzed (i.e., *n* = 10). The water permeability strengths were not correlated with those of glycerol (*r* = 0.02, Figure 3a), urea (*r* = −0.09, Figure 3b), and boric acid (*r* = −0.06, Figure 3c) permeabilities. The strength of glycerol permeability was weakly correlated with that of urea (*r* = 0.64, *p* < 0.05, Figure 3d) and boric acid (*r* = 0.59, *p* < 0.05, Figure 3e) permeabilities. The strengths of boric acid permeabilities were correlated with those of urea permeability (*r* = 0.995, *p* < 0.0001, Figure 3f).

**FIGURE 4:**
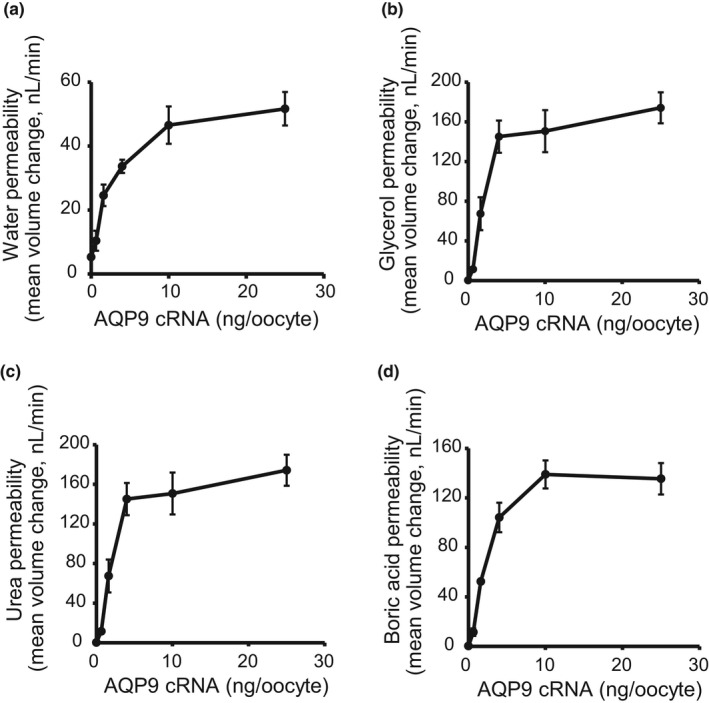
Effects of the amount of AQP9 cRNA injected into oocytes on water (a), glycerol (b), urea (c), and boric acid (d) permeability activities. Values for the results of the swelling assay are shown as mean ± SEM (*n* = 4–6)

For most experiments in the present study, we used oocytes injected with 25 ng of cRNAs for AQPs. The effects of the amount of injected cRNA were analyzed by the swelling assay using oocytes injected with 0.64, 1.6, 4, 10, and 25 ng cRNA for AQP9. The water, glycerol, urea, and boric acid permeabilities of AQP9 oocytes were elevated when the injected cRNA was increased from 0.64 to 4 ng, and plateaued after 4 ng (Figure 4).

### 3.3 Boric acid permeability of *Xenopus* oocytes expressing human AQPs evaluated by quantitative determination of whole‐cell boron content using inductively coupled plasma mass spectrometry (ICP‐MS)

**FIGURE 5:**
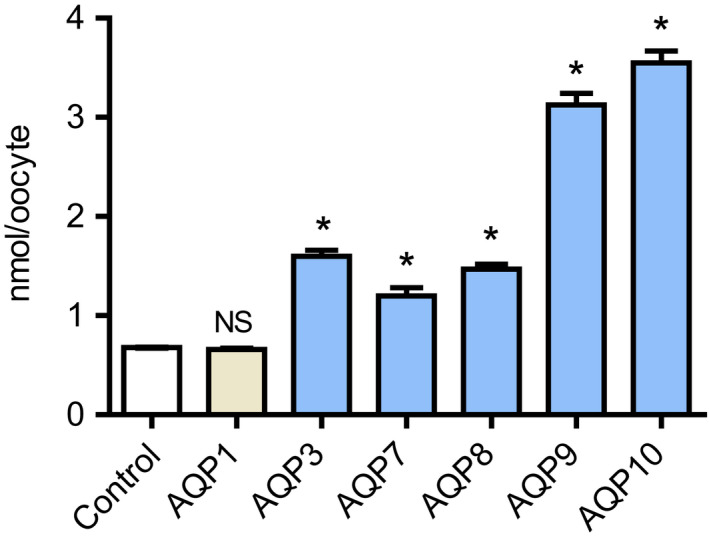
Boric acid uptake activity of AQP oocytes measured as whole‐cell boron content using ICP‐MS. Statistical significance was evaluated using the Kruskal–Wallis test followed by the Mann–Whitney *U* test; the Bonferroni correction was applied for multiple comparisons (*p < 0.05/6 = 0.0083). Values are shown as mean ± SEM (*n* = 6)

To directly confirm that exogenous expression of AQP3, 7, 8, 9, and 10 mediated boric acid influx, we next analyzed the whole‐cell boron content of oocytes expressing AQPs immersed in a solution containing boric acid and compared this with that of water‐injected control oocytes. After exposure to an iso‐osmotic solution containing 10 mM boric acid for 10 min, the whole‐cell boron content of oocytes expressing AQP3, 7, 8, 9, and 10 was significantly higher than that of control oocytes (*n* = 6 for AQP3, *p* < 0.0083; *n* = 6 for AQP7, *p* < 0.0083; *n* = 6 for AQP8, *p* < 0.0083; *n* = 6 for AQP9, *p* < 0.0083; *n* = 6 for AQP10, *p* < 0.0083; vs. control, *n* = 6 using the Mann–Whitney *U* test with Bonferroni correction *α* = 0.0083) (Figure 5). In contrast, the whole‐cell boron content of oocytes expressing AQP1 (*n* = 6), an orthodox AQP, was similar to that of control oocytes.

### 3.4 Boric acid permeability of *Xenopus* oocytes expressing human AQPs evaluated by electrophysiology

**FIGURE 6:**
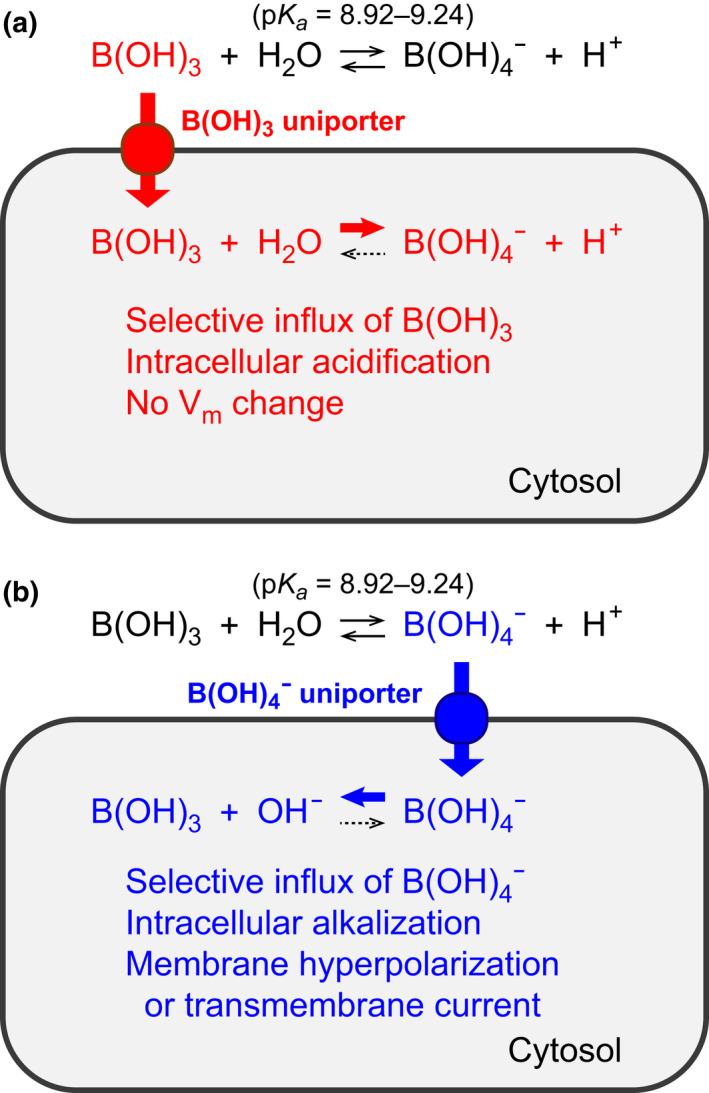
Hypothetical schematic diagrams of *Xenopus* oocytes expressing membrane protein with B(OH)_3_ or B(OH)_4_^–^ selective uniport activity. (a) Putative changes in intracellular pH (pH_i_) and membrane potential (*V*_m_) of an oocyte after the channel‐mediated selective influx of B(OH)_3_ are shown in red. In cytosol, the loaded B(OH)_3_ are partially reacted with water and converted into B(OH)_4_^–^ and H^+^. *V*_m_ will not change in response to the influx of B(OH)_3_. (b) Putative changes in pH_i_ and *V*_m_ of an oocyte after the channel‐mediated selective influx of B(OH)_4_^–^ are shown in blue. In cytosol, the loaded B(OH)_4_^–^ are partially converted into B(OH)_3_ and OH^–^. Membrane hyperpolarization or transmembrane current may be observed in response to the anion conductance

**FIGURE 7:**
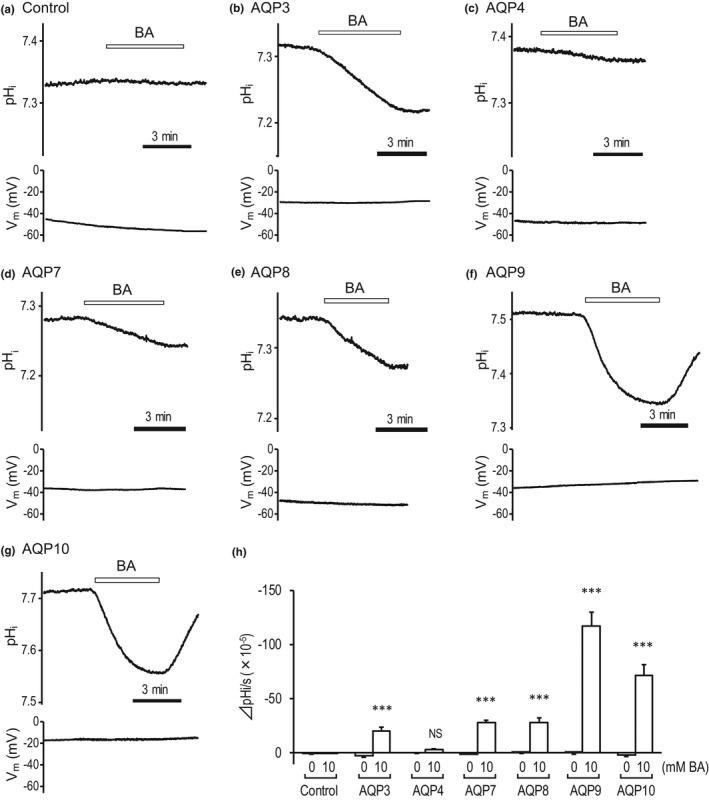
B(OH)_3_ channel activity of AQP3, 7, 8, 9, and 10. (a–g) Representative traces of changes in intracellular pH (pH_i_) and membrane potential (*V*_m_) of a control oocyte (a) and oocytes expressing AQP3 (b), AQP4 (c), AQP7 (d), AQP8 (e), AQP9 (f), or AQP10 (g). BA, 10 mM boric acid. (h) The summary of pH changes (dpH_i_/s) in control and AQP oocytes immersed in solution containing 0 or 10 mM boric acid. Values are shown as mean ± SEM (*n* = 4–11). Statistical significance was evaluated by the Welch's *t*‐test (***p < 0.05)

Boric acid (B(OH)_3_) is in equilibrium with borate (B(OH)_4_^–^) in aqueous solution (pKa 8.92–9.24) (Lopalco et al., 2020). To determine which form is transported in oocytes expressing AQPs, B(OH)_3_ or B(OH)_4_^–^, we analyzed changes in intracellular pH (pH_i_) of oocytes expressing AQPs in solution containing boric acid. As shown in Figure 6, B(OH)_3_ influx elicits intracellular acidification, whereas the influx of B(OH)_4_^–^ elicits intracellular alkalization and membrane hyperpolarization. Changes in pH_i_ were analyzed in oocytes expressing AQP3, 7, 8, 9, and 10 in iso‐osmotic solution containing 10 mM boric acid. Water‐injected oocytes and oocytes expressing AQP4 were used as negative controls. In oocytes expressing AQP3, 7, 8, 9, and 10, exposure to an iso‐osmotic solution containing boric acid resulted in a marked decrease in pH_i_ (*n* = 11 for AQP3, *p* < 0.001; *n* = 4 for AQP7, *p* < 0.001; *n* = 8 for AQP8, *p* < 0.001; *n* = 13 for AQP9. *p* < 0.001; *n* = 9 for AQP10, *p* < 0.001 using the Welch's *t*‐test), but did not elicit a change in membrane potential (Figure 7). No changes in pH_i_ were observed in water‐injected oocytes (*n* = 7) or AQP4 oocytes (*n* = 7). These results show that AQP3, 7, 8, 9, and 10 transport B(OH)_3_ and act as boric acid channels.

**FIGURE 8:**
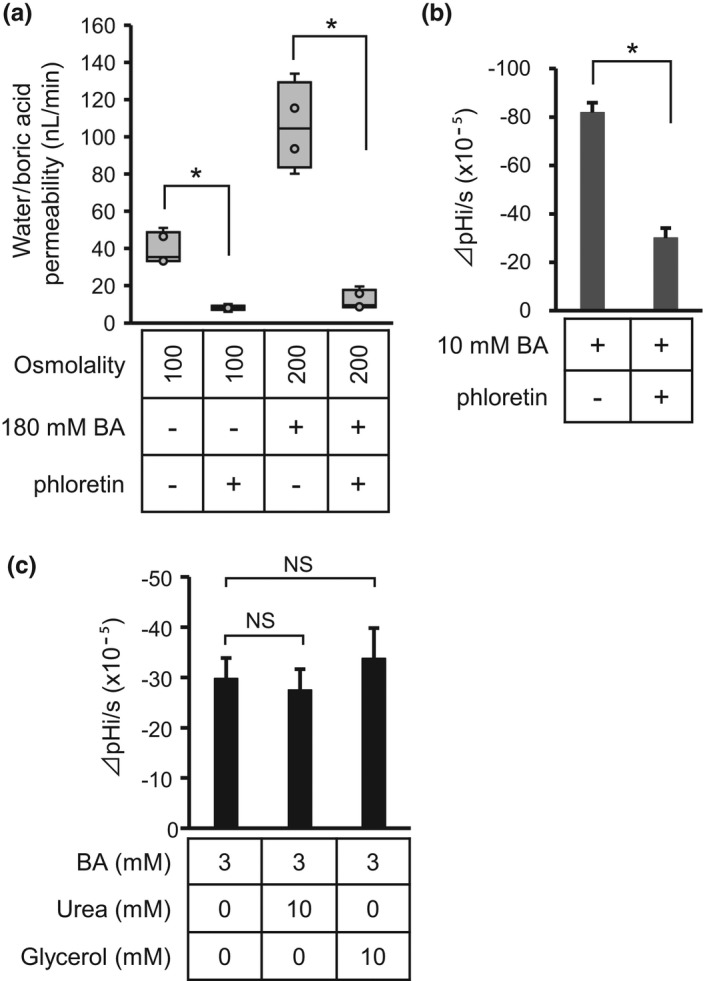
Effect of phloretin, urea, and glycerol on boric acid permeability of oocytes expressing AQP9. (a) Effect of phloretin on increase in volume of AQP9 oocytes in hypo‐osmotic solution or iso‐osmotic solution containing 180 mM boric acid. The changes in volume of AQP9 oocytes were analyzed by the swelling assay in the presence or absence of 100 μM phloretin. Values are presented as median (line), interquartile range (box), range (whiskers), and outliers (>1.5× interquartile range above upper quartile). Statistical significance was evaluated using the Mann–Whitney *U* test. *p < 0.05, *n* = 4–5. (b) Effect of phloretin on boric acid permeability of oocytes expressing AQP9. B(OH)_3_ permeabilities of AQP9 oocytes were evaluated by the changes in intracellular pH (ΔpHi/s) in solution containing 10 mM boric acid with or without 100 μM phloretin. Values are shown as mean ± SEM (*n* = 6), and statistical significance was evaluated using the Mann–Whitney *U* test. *p < 0.05. (c) Effect of urea and glycerol on boric acid permeability of oocytes expressing AQP9. B(OH)_3_ permeabilities of AQP9 oocytes were evaluated by ΔpHi/s in solution containing 3 mM boric acid with or without 10 mM urea or glycerol. Values are shown as mean ± SEM (*n* = 4), and statistical significance was evaluated using the Kruskal–Wallis test followed by the Mann–Whitney *U* test; the Bonferroni correction was applied for multiple comparisons (NS, *p* > 0.05/2 = 0.025)

Electrophysiological analysis revealed that B(OH)_3_ permeability was also detected as pH_i_ changes in AQP9 oocytes in iso‐osmotic solution containing 3 mM boric acid (Figure 8c). When 10 mM glycerol or urea was added with 3 mM boric acid in the iso‐osmotic solution, the B(OH)_3_ permeability, the decreasing rates of pH_i_ elicited by boric acid, were not significantly altered (Figure 8c).

### 3.5 Phloretin inhibition of boric acid permeability of *Xenopus* oocytes expressing AQP9

AQP9 activity is known to be inhibited by several reagents, including phloretin (Tsukaguchi et al., 1998). The inhibitory activity of phloretin on boric acid permeability was analyzed in oocytes expressing AQP9. The results of the swelling assay showed that phloretin significantly inhibited the increase in cell volume of AQP9 oocytes in both hypo‐osmotic solution and iso‐osmotic solution containing boric acid (*n* = 4–5, *p* < 0.05, Mann–Whitney *U* test) (Figure 8a). These results confirmed that phloretin inhibited the water permeability of AQP9 oocytes. The results of the electrophysiological analysis using pH microelectrode showed that boric acid‐elicited changes in pH_i_ in AQP9 oocytes were also significantly inhibited by phloretin (*n* = 6, *p* < 0.05, Mann–Whitney *U* test) (Figure 8b), indicating that phloretin inhibited B(OH)_3_ permeability in AQP9 oocytes.

### 3.6 Comparison of the pores of boric acid‐permeable and non‐permeable AQPs by computational structural analysis

**FIGURE 9:**
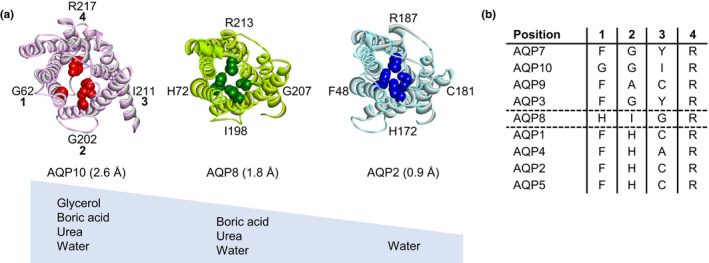
Comparison of the pores of boric acid‐permeable and non‐permeable AQPs by computational structural analysis. (a) Structures of the pore and the aromatic/arginine (ar/R) selectivity filter of AQP10, 8, and 2. The ar/R residues of AQP10 (PDB: 6F7H), and AQP8 (Model: AlphaFold‐Q94778), and AQP2 (PDB: 4NEF) at the positions 1, 2, 3, and 4 are represented by spheres. Each pore radius at the ar/R selectivity filter is shown in parentheses. Permeating molecules of each AQP are shown in the lower panel. (b) The ar/R residues of acid‐permeable and non‐permeable AQPs

The pore properties of boric acid‐permeable AQPs (AQP3, 7, 8, 9, and 10) and non‐permeable AQPs (AQP1, 2, 4, and 5) were compared using computational structural analysis. The estimated pore sizes of aquaglyceroporin AQP10 and AQP8 were larger than those of the orthodox aquaporin AQP2 (Figure 9a). The aromatic/arginine (ar/R) selectivity filters of boric acid‐permeable AQPs (AQP3, 7, 8, 9, and 10) are constructed of several small residues (G, A, I), whereas the ar/R residues at positions 1 and 2 in boric acid non‐permeable AQPs (AQP1, 2, 4, and 5) are large and highly conserved (F, H) (Figure 9b). Therefore, the pore sizes of the former seem to be larger than those of the latter. It should be noted that position 2 of AQP8 consists of small/middle residue (I), which might make them permeable to middle‐sized molecules (urea and boric acid). Additionally, AQP8 has middle‐sized polar residues (H) at position 1 and orthodox aquaporins also have polar residues (H) at position 2. This polar residue and pore size would affect the permeability of glycerol (Beitz et al., 2006).

## 4 DISCUSSION

In the present study, we showed that human AQP3, 7, 8, 9, and 10 act as boric acid transport systems when expressed in *Xenopus* oocytes. As previously reported by others (Azad et al., 2021; Ishibashi et al., 1998, 2002; Koyama et al., 1998; Kuwahara et al., 1997; Lee et al., 1996; Misaka et al., 1996; Moss et al., 2020; Preston et al., 1992; Sasaki et al., 1994), swelling assays showed that all mentioned human AQPs transport water across the plasma membrane. Boric acid permeability was observed in oocytes expressing AQP3, 7, 8, 9, and 10, but not in those expressing AQP1, 2, 4, and 5. Boric acid permeability was also confirmed by elemental quantification and electrophysiological analysis. The boric acid permeability of APQ9 oocytes was elevated by injecting increasing amounts of AQP9 cRNA, and was significantly inhibited by phloretin. Computational structural analysis suggested that the estimated pore sizes of boric acid‐permeable AQPs (aquaglyceroporins and AQP8: AQP3, 7, 8, 9, 10) seem to be larger than those of boric acid non‐permeable AQPs (orthodox AQPs: AQP1, 2, 4, 5). These results strongly suggest that aquaglyceroporins and AQP8 transport boric acid probably as channels, whereas the orthodox AQP family members, that is, AQP1, 2, 4, and 5, do not have the ability to transport boric acid. This is the first demonstration of a boric acid transport system in mammals. Consistent with previous reports of boric acid transport activities of the aquaporin superfamily in plants (NIPs, XIPs) (Ampah‐Korsah et al., 2016; Takano et al., 2006), yeast (Dur3, Fps1) (Nozawa et al., 2006), and *Trypanosoma brucei* (TbAQP2) (Marsiccobetre et al., 2017), some members of the aquaporin superfamily function as boric acid transport systems in a wide range of organisms. In oocytes expressing human AQPs, boric acid permeability was correlated with urea permeability and weakly correlated with glycerol permeability. However, 10 mM urea or glycerol did not competitively inhibit the boric acid permeability of AQP9 oocytes in a solution containing 3 mM boric acid. Further detailed analyses are required to understand the mechanism by which aquaglyceroporins and AQP8 enhance the membrane permeability of boric acid.

AQP8 has been classified as an orthodox or unorthodox aquaporin, but not as an aquaglyceroporin; AQP8 transports water but not glycerol when expressed in *Xenopus* oocytes and its transport profile has not been fully elucidated. The urea transport activity of AQP8 has not been consistently evaluated (Liu et al., 2006). From a comparative and evolutionary perspective, zebrafish (*Danio rerio*) have three paralogs for AQP8: DrAqp8aa, DrAqp8ab, and DrAqp8b (Tingaud‐Sequeira et al., 2010). DrAqp8aa and DrAqp8ab transport water and urea but not glycerol, whereas DrAqp8b transports water but not urea or glycerol. Considering the present results together with categorizations determined by Tingaud‐Sequeira et al. (2010), AQP8 can be categorized as a water and urea transporter that is distinct from both orthodox aquaporins and aquaglyceroporins.

Electrophysiological analysis showed that the intracellular pH was reduced but the membrane potential did not change when AQPs absorbed boric acid in *Xenopus* oocytes. These results indicate that AQPs transport boric acid as B(OH)_3_ but not as B(OH)_4_^–^. This is not surprising when we consider that B(OH)_3_ is a small neutral solute (formula weight, 62 g/mol; molecular radius, 2.573 Å), and aquaglyceroporins and AQP8 transport water and other small neutral solutes. Comparison of the structures of orthodox aquaporin and aquaglyceroporins showed that the pore size of orthodox aquaporin is narrower than that of aquaglyceroporins and acts as a barrier against glycerol permeation (de Mare et al., 2020; Gotfryd et al., 2018; Wang et al., 2005). Our results suggest that a similar mechanism also functions as a barrier against B(OH)_3_ permeation in orthodox aquaporins.

Although the in vivo functions of aquaporins in boric acid homeostasis have not been clarified, they can be speculated on by considering the distribution of each AQP. AQP3 is expressed in many human tissues (Mobasheri et al., 2005) and may be involved in boric acid homeostasis in various tissues. AQP9 is highly expressed in the liver and may be involved in the hepatic transport of boric acid. In humans, ingested boric acid is rapidly absorbed by the gastrointestinal tract (Murray, 1998). The intestinal epithelial cells express AQP7, 8, and 10 in the apical membrane and AQP3 in the basolateral membrane (Zhu et al., 2016), suggesting that these AQPs mediate boric acid absorption via the transcellular pathway. Boric acid is filtered by the glomeruli and eliminated through urine (Murray, 1998). The collecting duct, the final segment of the renal tubule, is known to express AQP2 in the apical membrane and AQP3 and 4 in the basolateral membrane (Nielsen et al., 2002). Because AQP2 does not conduct boric acid, the collecting duct may have low boric acid permeability, which is beneficial for boric acid excretion.

## CONFLICT OF INTEREST

The authors declare no conflict of interest. KU is an employee of Chugai Pharmaceutical Company Limited. The funder did not have any role in the study design, data collection and analysis, decision to publish, or preparation of the manuscript.

## AUTHOR CONTRIBUTIONS

Kazutaka Ushio, Erika Watanabe, Michael F. Romero, and Akira Kato conceived and designed the research; Kazutaka Ushio, Erika Watanabe, Takehiro Kamiya, Ayumi Nagashima, Tadaomi Furuta, Genki Imaizumi, and Akira Kato performed the experiments; Kazutaka Ushio, Erika Watanabe, Takehiro Kamiya, Tadaomi Furuta, Michael F. Romero, and Akira Kato analyzed the data; Kazutaka Ushio, Erika Watanabe, Takehiro Kamiya, Toru Fujiwara, Michael F. Romero, and Akira Kato interpreted the results of experiments; Takehiro Kamiya, Toru Fujiwara, Michael F. Romero, and Akira Kato supervised the experiments; Kazutaka Ushio, Erika Watanabe, Tadaomi Furuta, and Akira Kato prepared the figures; Kazutaka Ushio, Erika Watanabe, Tadaomi Furuta, Michael F. Romero, and Akira Kato drafted the manuscript; Kazutaka Ushio, Michael F. Romero, and Akira Kato edited the manuscript; Kazutaka Ushio, Erika Watanabe, Takehiro Kamiya, Toru Fujiwara, Michael F. Romero, and Akira Kato approved the final version of manuscript.
